# Accelerated parallel algorithm for gene network reverse engineering

**DOI:** 10.1186/s12918-017-0458-5

**Published:** 2017-09-21

**Authors:** Jing He, Zhou Zhou, Michael Reed, Andrea Califano

**Affiliations:** 1 Department of Biomedical Informatics, Columbia University, 168th Street, New York, 10032 NY USA; 2 Department of Systems Biology, 1130 St Nicholas Street, New York, 10032 NY USA; 3 Department of Computer Science, New York, 10027 NY USA

**Keywords:** GPU-ARACNE, Parallel computing, Regulatory networks, Mutual information, Gene expression dataset, CUDA

## Abstract

**Background:**

The Algorithm for the Reconstruction of Accurate Cellular Networks (ARACNE) represents one of the most effective tools to reconstruct gene regulatory networks from large-scale molecular profile datasets. However, previous implementations require intensive computing resources and, in some cases, restrict the number of samples that can be used. These issues can be addressed elegantly in a GPU computing framework, where repeated mathematical computation can be done efficiently, but requires extensive redesign to apply parallel computing techniques to the original serial algorithm, involving detailed optimization efforts based on a deep understanding of both hardware and software architecture.

**Result:**

Here, we present an accelerated parallel implementation of ARACNE (GPU-ARACNE). By taking advantage of multi-level parallelism and the Compute Unified Device Architecture (CUDA) parallel kernel-call library, GPU-ARACNE successfully parallelizes a serial algorithm and simplifies the user experience from multi-step operations to one step. Using public datasets on comparable hardware configurations, we showed that GPU-ARACNE is faster than previous implementations and is able to reconstruct equally valid gene regulatory networks.

**Conclusion:**

Given that previous versions of ARACNE are extremely resource demanding, either in computational time or in hardware investment, GPU-ARACNE is remarkably valuable for researchers who need to build complex regulatory networks from large expression datasets, but with limited budget on computational resources. In addition, our GPU-centered optimization of adaptive partitioning for Mutual Information (MI) estimation provides lessons that are applicable to other domains.

**Electronic supplementary material:**

The online version of this article (doi:10.1186/s12918-017-0458-5) contains supplementary material, which is available to authorized users.

## Background

Accurate and systematic reconstruction of gene regulatory networks (reverse engineering) represents a crucial step in the revealing of drivers and mechanisms presiding over both physiologic and pathologic phenotypes. Many computational approaches have been proposed for the reverse engineering of gene regulatory networks from large-scale gene expression profiles. Most of these require repetitively evaluating gene-gene interactions using mathematical methods such as Pearson/Spearman correlation [1], linear/LASSO regression [2], Bayesian dependence [3], Mutual Information/Conditional mutual informaiton [4, 5] and topological patterns [6]. Among them, ARACNE [7, 8] serves as one of the most widely applied reverse engineering algorithms by the scientific community and has been broadly experimentally validated. Regulatory network inferred by ARACNE is calculated based on information theory, further refined by a network pruning process called data processing inequality (DPI) theorem, which is to infer direct regulatory relationships between transcriptional factors and their target genes. ARACNE has been shown to be useful in reconstructing context-specific transcriptional networks in multiple tissue types [7, 9]. The inferred network and its further interrogation have stimulated several new algorithms, successfully unveiling key regulatory mechanisms in cancer [10, 11], as well as drug mechanism of action [12].

However, the explosion of available RNA-Seq datasets drives increasing demand of efficient network reconstruction algorithms. There is a call for algorithm optimization and alternatives of implementation on different platforms. The development of parallel computing systems based on multi- and many-core GPUs and cloud computing offers the promise to massively accelerate bioinformatics algorithms, especially the compute-intensive ones such as ARACNE, whose repetitive estimation of gene-gene functional relationship could be elegantly parallelized by utilizing multi-grid, multi-block and multi-thread GPU computing structure. The core calculation of ARACNE can follow the single instruction, multiple data (SIMD) paradigm, further exploiting current GPU architecture advancement. Previous works have shown great efforts to re-implement sequential computational algorithms into parallel versions. Lachmann et al. redesigned ARACNE using JAVA massive multithreading [13], but the algorithm requires very large memory usage. Misra et al. showed an Intel Xeon Phi coprocessor based implementation of network inference algorithm using fixed bandwidth [14]. This effort uncovered the promise of future parallel implementation of bioinformatics algorithm, whereas the requirement of specific Intel Xeon coprocessor limited its availability to scientific community.

**Fig. 1:**
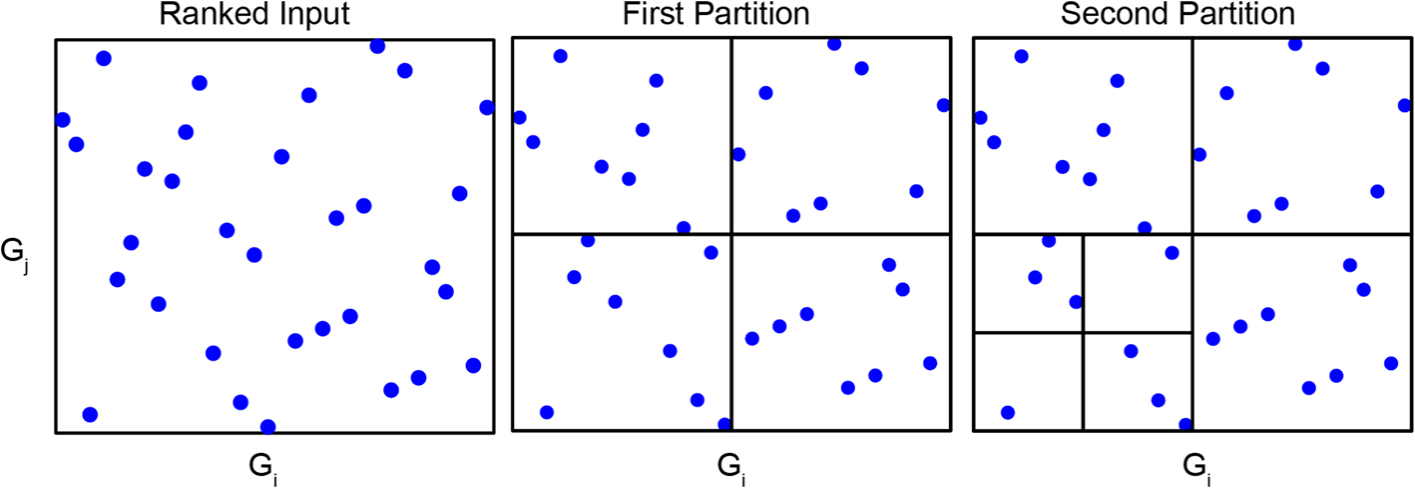
Adaptive partitioning schema to estimate mutual information. Each *blue point* represents one gene expression in one sample after ranking. X-axis and Y-axis represent 2 different genes. Boundaries are possible partitioning. *Left* plot shows the input data, *middle* plot shows the first partition, *right* plot shows a second partition

Yet, successful parallelized optimization while maintaining original estimation accuracy demands careful investigation of the algorithm, accurate approximation of data volume, proper estimation of hardware parallel capacity, and striking a fine balance between hardware and cross-platform applicability of the implementation. In view of this, a meticulous and thorough analysis of ARACNE was preformed to determine the possibility and magnitude of parallelism one can achieve by using the current GPU computing framework. In principle, ARACNE includes three major components: 1) Establishing the null model; 2) Computing a candidate network; 3) Pruning the network. Among them, computing the candidate network is the most computationally intensive step, requiring all potential pairs between transcriptional factors (TFs) and genes being calculated using MI, which is an information theory measurement of mutual dependence between two random variables. Thus, candidate interactions are identified by estimating Mutual Information (MI) of pairwise gene expression profiles *I*(*g*_*i*_;*g*_*j*_)=*I*_*ij*_). *I*_*i*_ *j* is zero if and only if joint probability density is the product of marginal probability densities, that is P(*g*_*i*_,*g*_*j*_)=P(*g*_*i*_)P(*g*_*j*_) MI exceeds other similar methods in its capacity to capture non-linear associations. Precisely, ARACNE uses an adaptive partitioning approach to estimate the joint probability, considering each partition’s statistical significance of providing enough information for further calculation, as outlined in Fig. 1. Based on this original design, current GPU computing framework could increase algorithm speed by orders of magnitude though largely parallelizing edge computing and MI estimation using multi-level threading. In addition, the null model building step would also likely be executed in parallel on separate GPU threads without costing additional time while the candidate network is being built. Furthermore, the availability of massively parallel threads in GPU could facilitate simultaneous pruning of candidate networks using thresholding and the Data Processing Inequality process (DPI, Eq. (1)) [7, 8].

1 $$\begin{array}{@{}rcl@{}}  I(g_{1};g_{3}) \leq \text{min}(I(g_{1};g2),~I(g_{2};g_{3})) \end{array} $$

Here, we present a complete redesign of ARACNE using GPU computing framework (GPU-ARACNE), mainly leveraging the performance of repeated MI estimation, network pruning using DPI, and statistics-based thresholding [15]. Our work shows a way to apply GPU intrinsic parallel capabilities to accelerate adaptive partitioning Mutual Information (apMI) calculation and simultaneously discovering pairwise interactions. GPU-ARACNE illuminates the potentiality of applying parallel computing techniques to solve computational or systems biology problems. We benchmarked the performance improvements of GPU-ARACNE using published TCGA breast carcinoma dataset [16] and prostate adenocarcinoma [17], and compared to previous versions [7, 13]. We reconstructed whole genome regulatory network using the NVIDIA CUDA framework, taking advantage of multi-core and multi-level parallelism, in which a hardware accelerator was designed to estimate apMI, innovatively exploiting concurrent access to GPU block shared memory to assist the estimation process. Furthermore, GPU-ARACNE encapsulates the three sequential steps once executed one after another into one step, simplifying overall workflow. GPU-ARACNE is attractive in its wide availability to researchers having GPU or having access to Amazon Web Service (AWS), and the generalizability of its optimization techniques (such as random shuffle and mutual information computation).

## Methods

This section starts with an overview of GPU-ARACNE workflow. Then we present how we implement the adaptive partition Mutual Information (apMI) estimation using GPU computing framework. In addition, we will discuss parallelized null model computation and parallelized network pruning respectively. Finally, the datasets used and hardware configuration will be listed.

***GPU-ARACNE workflow***

**Fig. 2:**
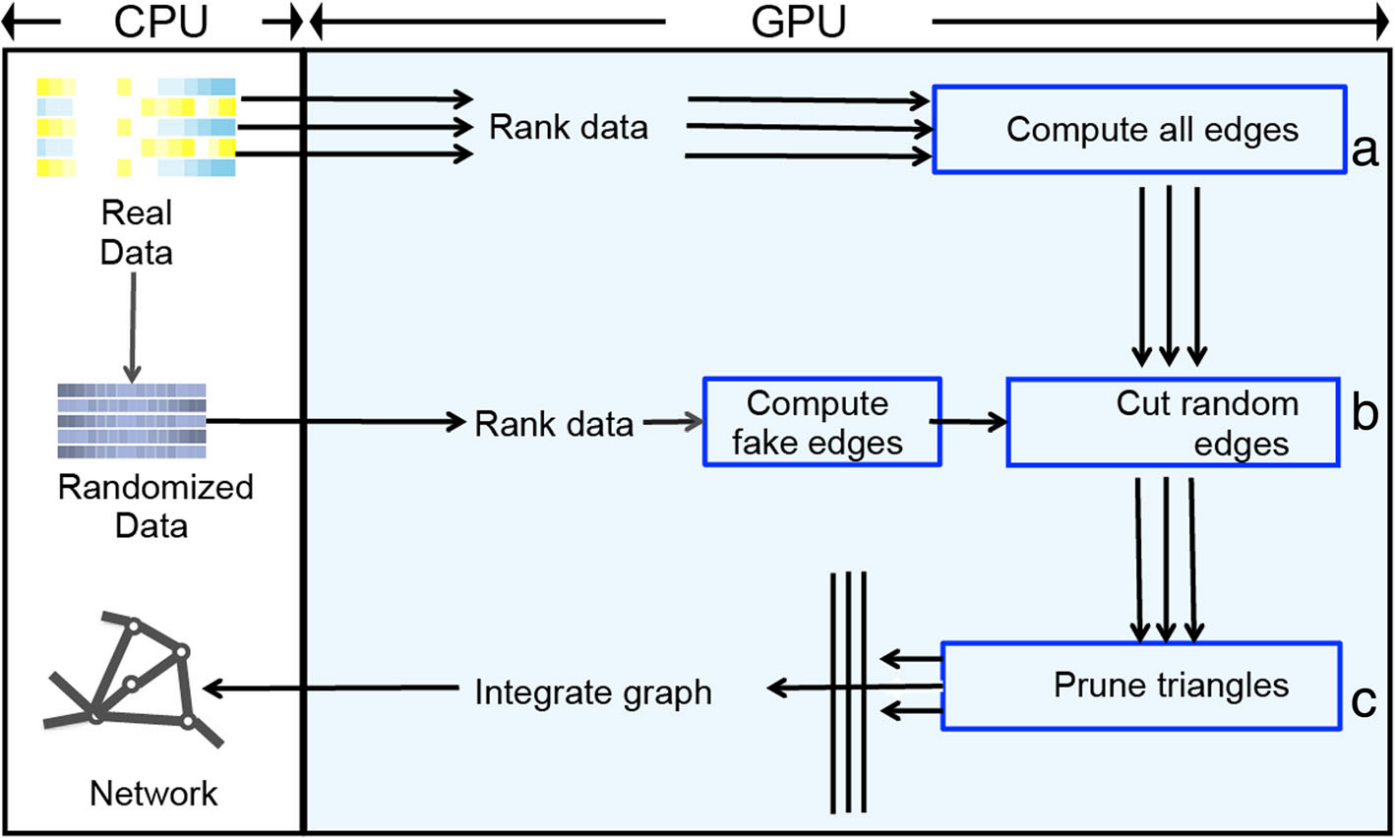
GPU-ARACNE workflow. *Left* box represents data/operations on host, and *right blue* box covers data/operations on GPU device. *Arrows* represent data flow. In *left* box, heat-map represents gene expression matrix and permuted matrix

We provided GPU-ARACNE, an accelerated parallel implementation to build regulatory networks on GPU computing frameworks. Based on GPU computing capability differences, two separate implementations were provided: GPU-ARACNE-V1 for GPU card with a 3.5 or higher compute capability and GPU-ARACNE-V2 for those with less than 3.5. For both versions, GPU-ARACNE was based on intrinsic parallelism of the original network reconstruction algorithm, ARACNE [7, 8] or ARACNE-AP [13]. All performance sensitive parts of the algorithm, including null model computation, apMI estimator, and the DPI process, were parallelized using GPU multi-core and multi-threading computing framework. We depicted the GPU-ARACNE workflow, crafted to illustrate the parallelism and our optimization efforts, in Fig. 2. At least three optimization efforts were achieved in this workflow: a). Using parallel computing to replace serial computing of all edges. Computing candidate edges in a network were independent events, which were highly paralleled in GPU-ARACNE instead of serial looping through all potential pairs one by one for both null model and real model in original version (Fig. 2 a). b). Parallel thresholding. Pruning the network edges, including thresholding edges and pruning triangles, are mutually independent events, which could be processed by different computing units in GPU, instead of by waiting to be sequentially processed in the serial version (Fig. 2 b–c). c). Parallelized apMI estimator was designed to replace the original MI estimation which was based on recursions.

***Paralleled adaptive partition mutual information estimation***

All candidate edges are calculated using adaptive partitioning MI (apMI, Eq. (2)) [13], a non-linear measurement of mutual dependence between two random variables. *x*_*i*_ is the expression of gene *x* in sample *i*, *y*_*i*_ is the expression of gene *y* in sample *i*, *N* is the MI normalization factor. *f*(*x*_*i*_,*y*_*i*_ denotes the joint density, while *f*(*x*_*i*_) and *f*(*y*_*i*_) represents the marginal probability density before normalization, respectively.

2 $$\begin{array}{@{}rcl@{}}  \text{MI}_{x,y} = I(x;y) = \frac{1}{\mathrm{N}}\sum_{i}\text{log}\frac{f(x_{i},y_{i}}{f(x_{i})f(y_{i})} \end{array} $$

**Fig. 3:**
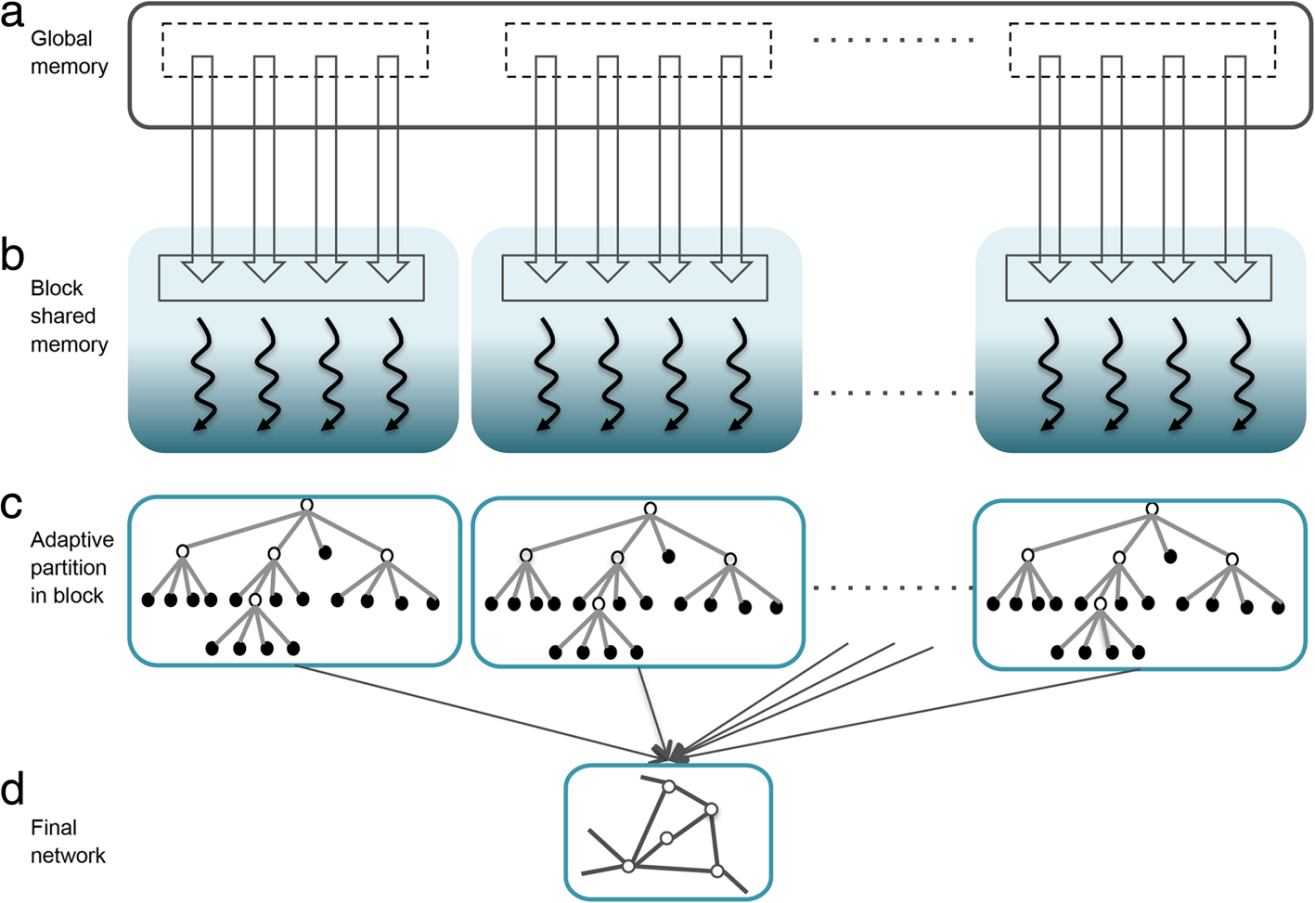
GPU multi-threading structure schema. **a** Data/operations on GPU global memory. *Dotted boxes* represent virtual warps. **b** In GPU block shared memory, each *blue shaded box* represents one GPU block. Each *curved line* represents one thread. **c** Stored data-structure to compute mutual information for each pair of genes. One node in the tree represents un-normalized MI calculated from one quartz after each partitioning, stored in each block shared memory. **d** Network after collecting all pairwise mutual information

**Optimized ranking** As shown in Fig. 2, after data was transferred to the GPU, threads were immediately launched, with each of them responsible for ranking one gene, similar to what has been done in previous work [18]. Precisely, each thread called a specific rank function from thrust, a library for CUDA computing framework [19]. This ranking process was optimized by splitting all data points into tablets, saving the ranked data on GPU global memory (Figs. 2 a and 3 a).

**Optimized MI computing** Subsequently, a 2D kernel indexed by TF-target pairs was launched to compute pairwise MI (Fig. 3 b). Strictly, one thread was responsible for one data point in partitioning, executing one binary comparison to decide which quartz the data point belongs in current partitioning, and then storing the results in an imbalanced tree data structure in block shared memory (Figs. 1, 3 b–c). However, this imbalanced tree structure shared by different threads brought up threads divergence within one block, severely harming parallelism. Thus, we innovated a breadth-first-searched tree using a queue structure, minimizing threads divergence (Fig. 3 c), keeping the queue in block shared memory to record the state of adaptive partitioning and to count data points, respectively. As an intrinsic innovation, this design speeded up overall performance by leveraging the fast access and ever-increasing size of GPU block shared memory. In practice, we ensure that the calculation would be done in a way that once an observation vector *G*_*i*_ is loaded in the global memory of a GPU, all (*G*_*i*_,*G*_*j*_ pairs would be processed on the same GPU block. As the hyper-threads on a GPU share one global memory, we assign them to work on the same *G*_*i*_ and different *G*_*j*_ observation vectors using different block, along with different shared memory.

Analytically, the computation required for each (*G*_*i*_,*G*_*j*_) pair is independent, thus, we employ coarse-grained parallelism with each 2D block processing one TF-Target pair. This parallelism is even boosted in GPU-ARACNE-V1, where dynamic parallelism is available, allowing more threads being called by the threads in current GPU block using fine-grained parallelism. Practically, for each regulatory genes, its mutual information with all the other genes are calculated simultaneously based on the number of blocks and block-wise threads available for specific GPU card.

To be noticed, as the queue data structure is located in block shared memory, a careful calculation is essential for an optimal parallel execution. Theoretically, the queue structure is upper-bounded by sample size M - 3, and the maximum sample size this optimization can handle is bounded by the number of threads allowed to launch for each block, 1024 for the GPUs we used in this work.

***Null model for MI thresholding***

Null model was built by computing MI using randomized expression matrix *G*, which was permuted on CPU and then transferred to the GPU global memory (Fig. 2 a). Following, *N*_0_ MI values (default 100,000) were calculated as the baseline MIs, resulting in an empirical cumulative mass function which was then used to computed a null model MI cutoff at a given *p*-value level.

***Paralleled thresholding and data processing inequality***

All candidate edges computed in apMI step were subjected to two network pruning approaches: null model thresholding and DPI process. In null model thresholding step, N_tf_×N_target_ threads were invoked simultaneously where N_tf_ is the number of transcription factors and N_target_ is the number of target genes, masking all candidate edges instantly using MI threshold. Thresholding all candidate edges was implicitly applied to previous apMI result. The resulted candidate network was then saved in a Boolean matrix mask. In our practice, this step was done efficiently without a noticeable cost of memory.

While applying DPI on each TF-TF-Target triangle, a 2D kernel function indexed by an adjacency network matrix was launched. Explicitly, each thread operated on triangle to label the retaining edges, resulting in a Boolean matrix. This network mask was then saved in a Boolean matrix and subject to later operation. Technically, a total of N_tf_×N_target_ threads were launched on GPU to process all possible triangles. Here, we ignored all self-interactions, aka. The degenerated triangle was left without special consideration to reduce thread divergence.

***Paralleled bootstrapping and consolidation***

Bootstrapping was integrated into the overall workflow except being specified to run without bootstrapping. We achieved paralleled bootstrapping by initiating two additional matrices on CPU: one for the sum of all MIs for all bootstraps, the other for counts of occurrence of edges across all bootstraps. Final edges identity and occurrences were calculated by using parallel reduce function provided by thrust library [19]. These two bootstrap-related matrices along with the total edges and occurrence were shipped back to CPU after each bootstrapping.

Using the edge and occurrence information, a Poisson model was used to calculate the statistical significance of an edge to be claimed in the final network on CPU. Finally, after Bonferroni correction, an edge would be claimed and its corrected Poisson *p*-value would be output. This whole bootstrapping process was mostly done by leveraging data parallelism using different instance of randomly bootstrapped matrix *G* ^′^.

***Datasets and parameters***

Gene expression datasets were downloaded from The Cancer Genome Atlas (TCGA) [20], including Breast Invasive Carcinoma profiles (BRCA, *n* = 1152) and prostate adenocarcinoma expression profiles (PRAD, *n* = 550). TCGA gene expression was originally measured using RNA-seq and then normalized by the standard pipeline [16]. The data was subjected to standard TCGA quality control, cross-experimental normalization, etc.

We used two datasets to benchmark GPU-ARACNE-V1, 200 random BRCA samples expression and all 550 PRAD samples expression. A list of N_tf_=1788 genes annotated as TFs by previous work was used [7, 8]. A *p*-value threshold of was set for all runs of GPU-ARACNE-V1 and comparative algorithms.

For both BRCA and PRAD samples, we sub-selected samples and genes (1100 genes) to measure different algorithms run-time. For BRCA, we sampled expression matrix with 100, 200, 300, 400 and 500 samples. For PRAD, we sampled expression matrix from 100 to 550 samples, increasing by 50 samples, resulting in matrices of 1100×N_sample_, where N_sample_ is the number samples. We chose a small set of genes to make it practical to reveal the time complexity difference among algorithms. Theoretically, the speed changing trend should hold at any given gene number. We used 10 bootstraps and a *p*-value threshold of 10^−3^ for all runs of GPU-ARACNE-V2 and comparative algorithms.

***System configuration***

GPU configurations to run GPU-ARACNE-V1 and GPU-ARACNE-V2 were shown in Table 1. The source code is available from [Additional file 1]. CPU configuration to run all other implementations was listed in Table 2. During benchmark, GPU-ARACNE-V1 was compared with stand-alone CPU, which was the best CPU configuration we had access to. GPU-ARACNE-V2 was compared with AWS g2 instance CPU, which had the comparable configuration as for GPU-ARACNE-V2. Admittedly, given that the GPU and CPU are fundamentally different hardware systems, the configurations to run GPU-ARACNE-V1 might not be the best comparable setting, but they do resemble the computing resources of wide accessibility, which still matches of each other.

**Table 1:** GPU configuration

Parameter	GPU-ARACNE V1	GPU-ARACNE V1	GPU-ARACNE V2
	(AWS g2)	(AWS p2)	
GPU card name	GeForce GTX 780	K80	Kepler GK104
CUDA cores	1536 (192 ×8 MP)	2496	2304 (192 ×12 MP)
GPU clock rate (GHz)	0.9	0.72	0.8
Memory clock rate(MHz)	3004	2500	2500
Global memory (MB)	3072	12288	4096
Shared memory per block (KB)	48	48	48
Max. No. threads per block	1024	1024	1024

**Table 2:** CPU configuration

Parameter	Stand-along CPU (V1)	AWS CPU (V2)
CPU model number	Intel Core i5	Intel Xeon E5-2670 Processors
Memory (GB)	8	15
Max. memory bandwidth (GB/s)	25.6	51.2
The number of CPU cores	2	8
The number of threads	4	16

## Result

### Time complexity analysis

Accounting for compute capability difference between GPU computing frameworks, we provide GPU-ARACNE-V1 for GPU card with 3.5 or higher compute capability, GPU-ARACNE-V2 for GPU card with 3.5 or lower. GPU-ARACNE-V1 runtime was measured using both NVIDIA GeForce GTX 780 (stand-alone card) and K80 card installed on AWS p2.xlarge EC2 instance. GPU-ARACNE-V2 performance was measured using Kepler GK104 card deployed on AWS g2.xlarge EC2 instance.

#### GPU-ARACNE-V1 runs faster than other implementations

**Fig. 4:**
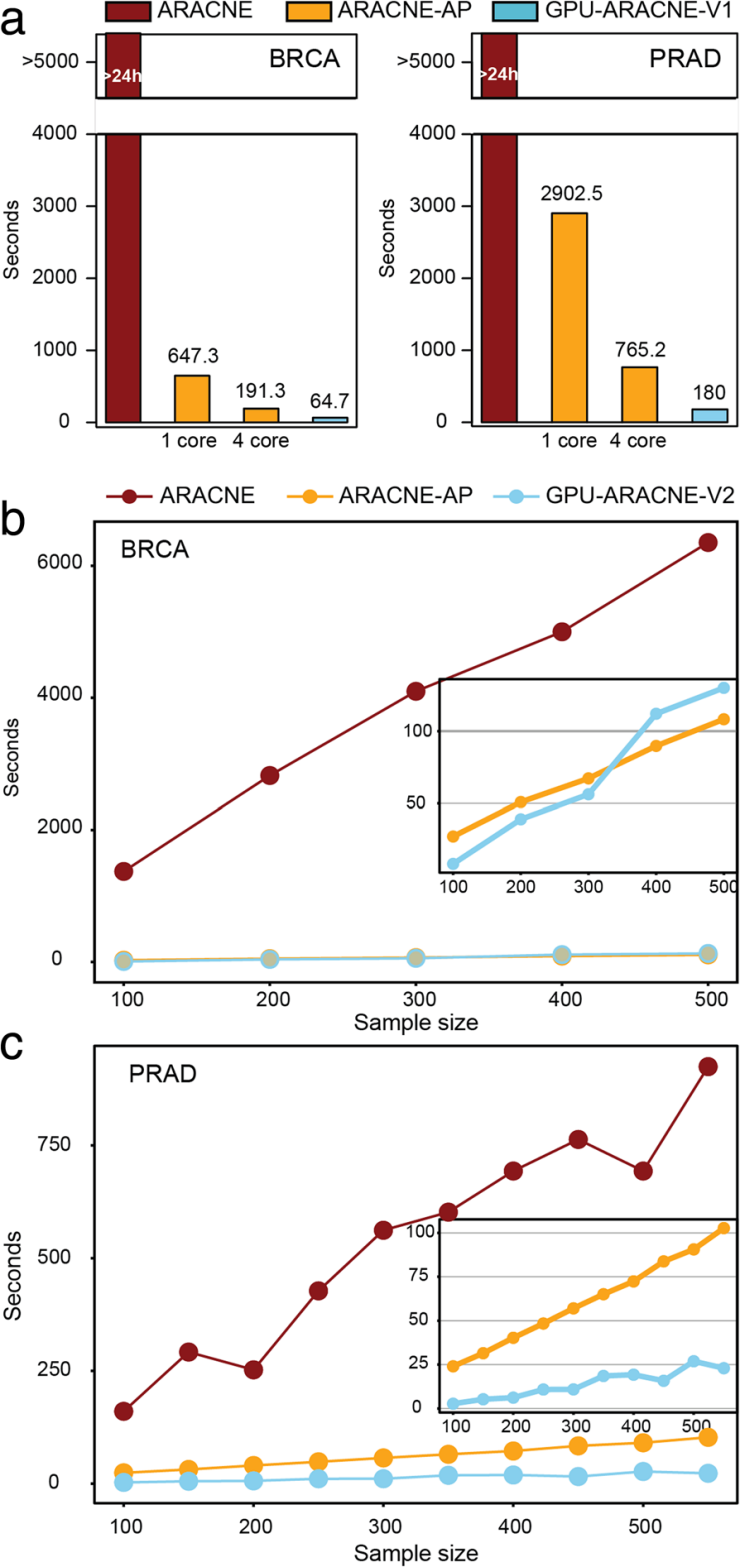
GPU-ARACNE speed benchmarking. **a** GPU-ARACNE-V1 performance. X-axis represents different algorithms/setting. Y-axis represents runtime in seconds. ARACNE runtime is truncated to show ARACNE-AP and GPU-ARACNE-V1 running time. *Left plot* shows result using 200 breast cancer samples, *right plot* shows result using 550 prostate cancer samples. **b**-**c** GPU-ARACNE-V2 performance. X-axis represents sample size; Y-axis shows the running time in seconds

GPU-ARACNE-V1 was developed on a high-end GPU card supporting Dynamic Parallelism, enabling additional threads launching in current kernels. In this work, we had access to two such high-end NIVIDIA GPU cards: GTX-780 and K80 deployed on AWS p2.xlarge GPU instance. Performance of GPU-ARACNE-V1 was measured on both GPUs, using BRCA and PRAD datasets. As a comparison, we also ran ARACNE [7, 8] and ARACNE-AP [13] using the same data with comparable CPU configuration. GPU-ARACNE-V1 was shown as the fastest implementation compared to ARACNE and ARACNE-AP for both BRCA and PRAD datasets (Fig. 4 a). In either case, ARACNE, the first implementation, took more than a day. ARACNE-AP achieved at least 8X improvement by using JAVA multi-threaded parallelism, 1-core or 4-core. In addition to JAVA parallelization, GPU-ARACNE-V1 further reduced the running time by a factor of at least 3, reducing runtime to 1 min for BRCA dataset, to 3 m for PRAD dataset. This advancement of GPU-ARACNE-V1 comparing to ARACNE-AP was shown to be further extended to 4.2X in PRAD dataset where sample size was increased to 550. Thus, we could conclude that GPU-ARACNE-V1 is relatively faster compared to all available ARACNE implementations.

There is possibility that using more CPU cores would boost ARACNE-AP performance over GPU-ARACNE-V1. In our work, given the same memory, ARACNE-AP ran on 4 cores overused CPU memory such that we did not observe a 4 times speed increase comparing to 1 CPU core run. Thus, it’s fair to assume ARACNE-AP run with 4 CPU cores already reaches its high performance at given memory size. With increased memory, CPU with additional cores would definitely help ARACNE-AP achieve faster speed, but more advanced GPU card can also improve GPU-ARACNE performance. Another practically challenge for ARACNE-AP is that, most machines using CPU for other works whereas running ARACNE-AP would exhaust available system memory, making it impossible to work simultaneously with other tasks. This is not the case for GPUs who are mostly idle for system tasks, making it economical to run GPU-ARACNE.

#### GPU-ARACNE-V2 runs relatively faster than other implementations

To further take advantage of idle GPU resource, we provided GPU-ARACNE-V2 for GPUs with compute capability less than 3.5. We used NVIDIA GPU Kepler GK104 installed on AWS g2.xlarge instance to benchmark GPU-ARACNE-V2 performance. To reveal the time complexity difference among ARACNE, ARACNE-AP, and GPU-ARACNE-V2, we used subsets of BRCA and PRAD dataset for all runs, with same *p*-value and bootstrapping number. As a result, GPU-ARACNE-V2 and ARACNE-AP showed an overall superiority to ARACNE for both BRCA and PRAD data, with at least 30X runtime decreasing in BRCA and minimal 10X decline in PRAD (Fig. 4 b).

In between the two parallel algorithms, GPU-ARACNE-V2 was at least comparable to ARACNE-AP. Executing on BRCA dataset, their runtimes were similar. GPU-ARACNE-V2 performed better than ARACNE-AP at start, then was surpassed by it when sample size climbed over 300. However, ARACNE-AP’s occasional superiority was gained without considering the system memory usage, which was a big factor for most JAVA programs. Naturally, we speculated that this runtime difference might lie in the available memory difference between GPU and CPU. In this case, the AWS g2 instance had 8G CPU memory but only 4G GPU memory. The stable growing of ARACNE-AP runtime might take advantage of polynomial increased CPU memory usage. This assumption was then experimentally proved when we controlled the available CPU memory for ARACNE-AP when running PRAD dataset, showing that GPU-ARACNE-V2 was always superior to ARACNE-AP. Even though both GPU-ARACNE-V2 and ARACNE-AP employed a linear runtime increasing as sample size growing up from 100 to 550, GPU-ARACNE-V2 did grow slower than that of ARACNE-AP. One point to be noticed was that, as the sample size for all datasets tested in this work were smaller than 1024, which is the GPU hardware limitation for optimal parallelism, we would expect the above mentioned runtime growth trend being held. Thus, we could conclude that ARACNE-GPU-V2 ran faster compared to other implementations with no more than 1024 samples.

We further explored algorithm scalability regarding null model *p*-value and bootstrapping number. As regulatory network reconstruction was usually done in whole genome, it is legitimate to compare regulatory network reconstruction algorithms while fixing gene number as a constant. With sample size being held, varying null model *p*-value would influence network reconstruction network pruning steps. For example, a larger *p*-value would result in more edges being kept as candidate edges, accumulating more triangles in DPI step. While in GPU-ARACNE, as all triangles were evaluated concurrently by different threads, a larger *p*-value and its consequences would not harm overall performance, in contrast to all the previous implementations [7, 13], whose runtime would be increased as a result of sequential cutting and serial DPI. However, increased bootstrap number does degrade the performance of all algorithms. In GPU-ARACNE, the increased run time was mostly due to data transfer between host and GPU device. The serial data shipping could be solved when multiple GPU devices are available, such as in GPU cluster in Amazon Elastic Compute Cloud, where different bootstraps could be executed in parallel on different GPUs. Otherwise, the limited availability of GPU devices will force the algorithm to revert to serial mode with bootstraps. Such was the case for almost all algorithms in this kind.

Another factor in evaluating algorithm performance is memory usage. Theoretically, the memory requirement of GPU-ARACNE is proportional to the size of data matrix. While for ARACNE-AP, besides that larger dataset requires more memory to hold, the number of threads needed in JAVA Virtual Machine (JVM) also scale up with data size. Therefore, since JAVA threads need basic stack memory (typically 1024 KB per thread), larger data size would require significantly more memory to be allocated to JVM. Taking a simple example, an allocation of 2 GB memory size for JVM can support maximally 2000 threads, indeed limiting the level of parallelism during execution.

In summary, GPU-ARACNE-V2 definitely ran faster than ARACNE, and at least comparable to multi-threading ARACNE-AP. Within the hardware limitations, the runtime scaled linearly regarding to samples size in our experiments.

### Accuracy analysis

We evaluated GPU-ARACNE accuracy by demonstrating consistency of estimated MI, and then showed network structure validity.

#### MI consistency

**Fig. 5:**
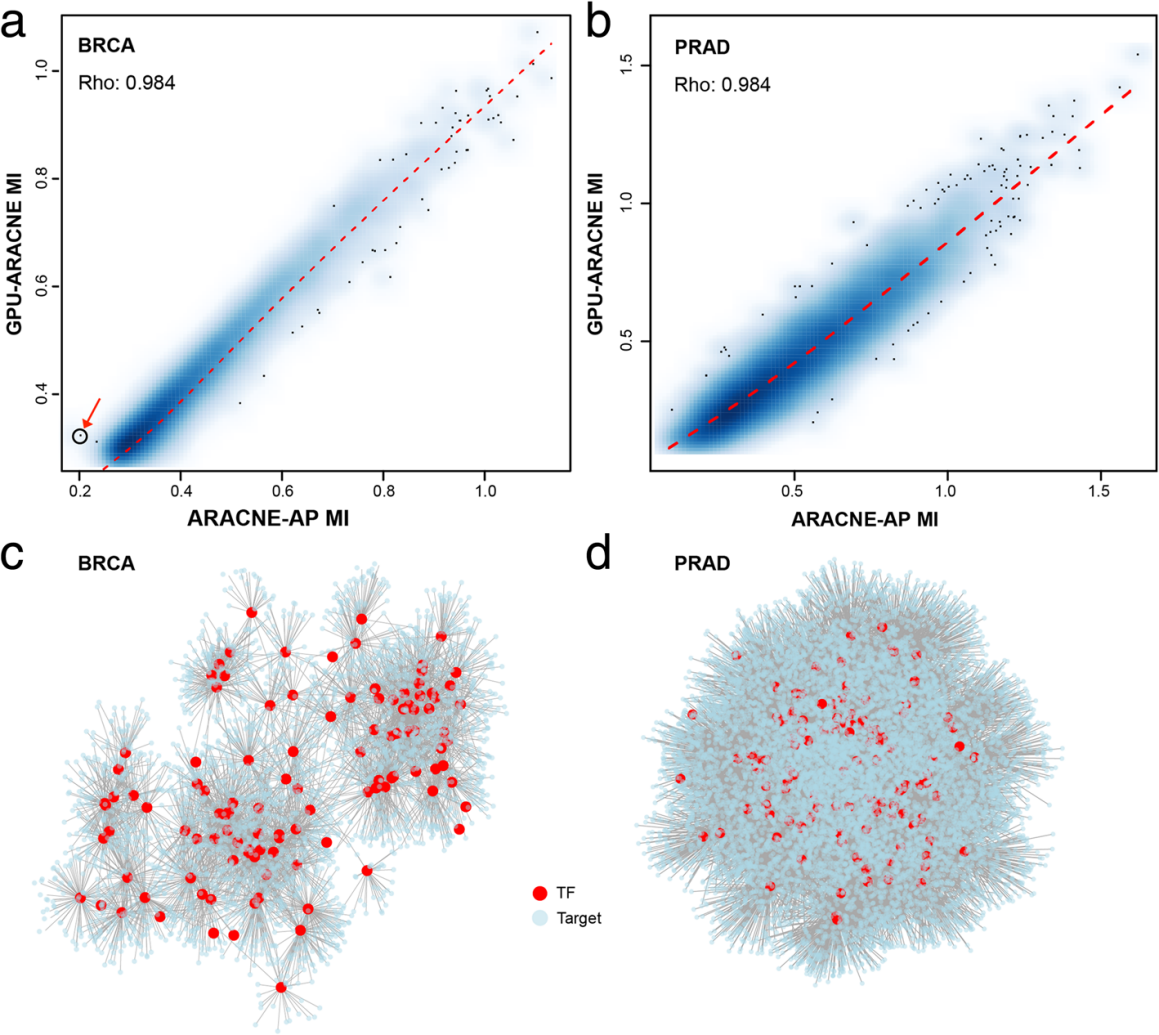
Estimated mutual information and networks. **a**-**b** Density plot of MI calculated from GPU-ARACNE and ARACNE-AP for breast cancer and prostate cancer dataset, respectively. X-axis represents ARACNE-AP MI; Y-axis represents GPU-ARACNE MI. Each *black points* represent one pairwise MI in corresponding gene regulatory network. The *blue shades* correspond to density of points representing pairwise MIs. **c**-**d** Subnetwork of breast cancer and prostate cancer gene regulatory network of top 100 most connected transcription factors and their targets

As Lachmann et al. had shown that ARACNE-AP MIs were consistent with that from ARACNE [13], we chose to focused on comparison between GPU-ARACNE MIs and ARACNE-AP MIs to evaluate GPU-ARACNE estimated MI accuracy. The networks used in this comparison were reconstructed using identical *p*-value and bootstrapping settings for both ARACNE-AP and GPU-ARACNE (*p*-value: 10^−8^, no bootstrapping). In general, MIs from GPU-ARACNE were significantly correlated with that from ARACNE-AP (*ρ*=0.984, for BRCA and PRAD, Fig. 5 a-b), indicating consistent edge weights computed from both algorithms.

Still, there were cases where the two algorithms’ MI inference showed deviance (Fig. 5 a–b, black dots). For some gene pairs, ARACNE-AP predicted lower MI, while GPU-ARACNE over-estimated it. For example, one interesting observation was the edge between gene pair LEF1 (Lymphoid Enhancer Binding Factor 1, the transcription factor [21]) and LRRC15 (Leucine Rich Repeat Containing 15 [22]) (Fig. 5 a, red arrow). LEF1 enhancer facilitated Wnt pathway in tumor invasion [23], while LRRC15 protein, which was usually over-expressed in tumor, particularly breast tumors [24], was reported as a potential drug target for virus based cancer therapy [25]. This interaction provides valuable information for molecular mechanism to understand LRRC15 related treatment. In this work, GPU-ARACNE detected such regulation confidently, contrasting to ARACNE-AP where the inferred interaction strength was only at borderline. On the other hand, GPU-ARACNE tends to underestimate the MIs when their values become larger (Fig. 5 a, top right). This might result from two factors: 1) a deeper searching tree in the GPU apMI estimator where more information was calculated comparing to other implementations; 2) a different arithmetic approximation of floating point in GPU comparing with CPU. Despite of all those differences, GPU-ARACNE network employed a structure that was almost identical to that from ARACNA-AP. Moreover, as all network pruning process was based on lower bound MI value, which was not affected by the underestimation of higher MI pairs. GPU-ARACNE reconstructed network was at least as accurate as ARACNE-AP, with a possibility of slightly increased sensitivity.

Analysis of the sub-network composed of the top 100 most connected TFs and their targets in both BRCA and PRAD data showed densely connected context-specific networks (Fig. 5 c–d). In BRCA sub-network, TFs had an average degree of 53.95, while the top connected TFs such as Forkhead box protein M1 (FoxM1), SOX10, had more than 100 inferred targets. The sub-network connectivity was even increased in PRAD, whose top 100 connected TFs sub-network had an average degree of 178.21. The increased connectivity might be resulted from larger samples size that enabling more perturbations of gene regulation, also the context specific property of GPU-ARACNE networks.

#### FoxM1 regulon

**Fig. 6:**
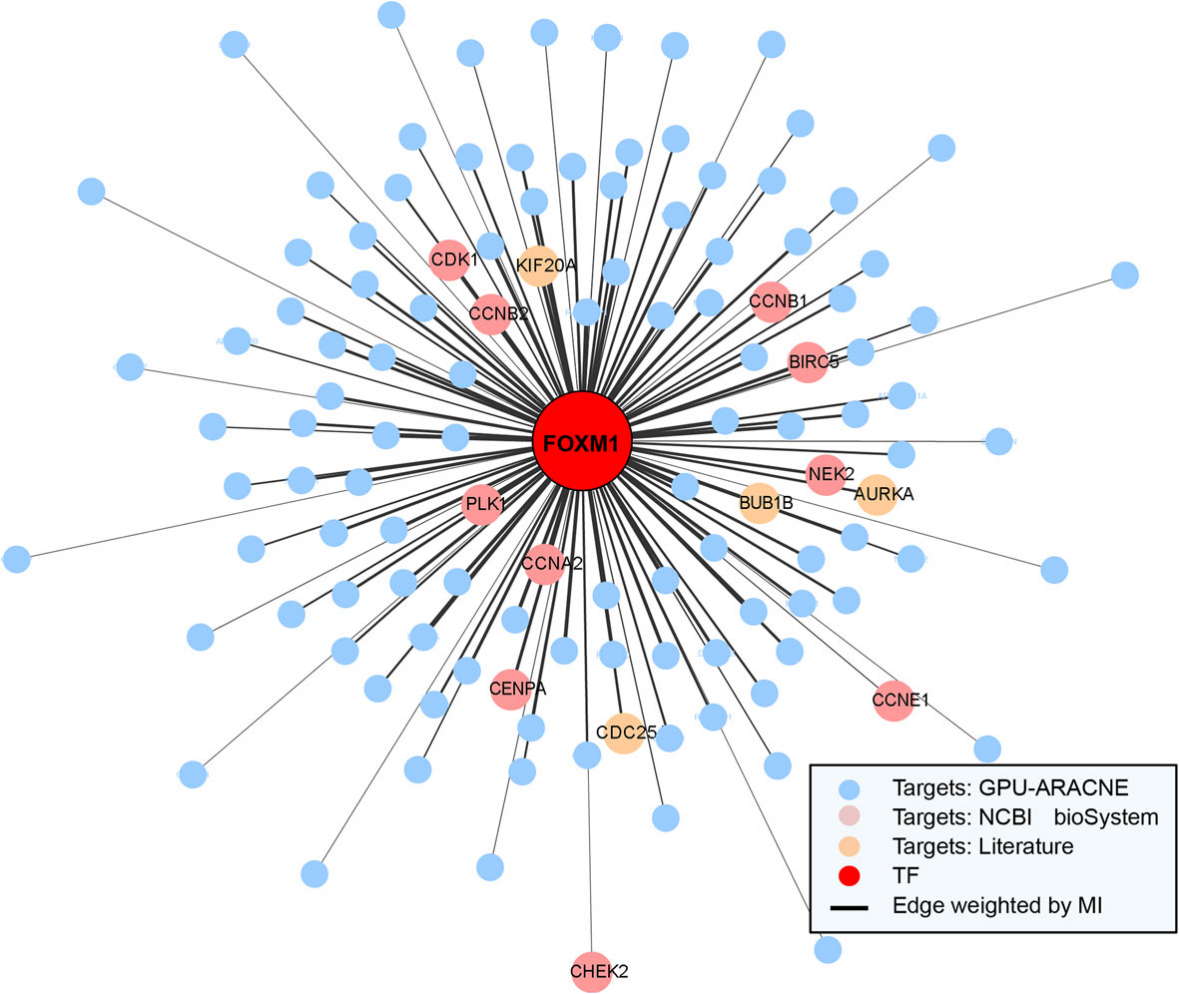
FoxM1 breast cancer regulon. GPU-ARACNE predicted FoxM1 breast cancer specific regulon, centered by FoxM1 as the transcription factor, connecting to its predicted transcriptional gene targets (other circles). FoxM1 targets were color coded according to the evidence validating their biological relevance. NCBI curated database reported targets were colored as *pink* ones; Literature reported by experiments were colored as *orange* ones. All edge length and thickness were weighted by the estimated Mutual information between targets and FoxM1. The closer one target is to FoxM1, the stronger the interaction is

To further understand the biological meaning of GPU-ARACNE inferred gene regulatory network, we focused on breast cancer regulatory network, specifically FoxM1 (Forkhead box protein M1) transcriptional network (regulon) as FoxM1 was predicted to have the largest regulon by GPU-ARACNE (Fig. 6), supporting previous findings that it was highly activated in breast cancer tumors [26, 27]. Moreover, FoxM1 was reported as a master regulator and a biomarker in breast cancer by multiple studies [28–31]. The function of FoxM1 was believed to be widely associated with cell proliferation [32], mitosis [33], and DNA damage [34]. Also, previous work in lab has experimentally validated the regulatory role of FoxM1 in different contexts [9, 10]. To validate the prediction accuracy of targets of TFs, we focused on finding biological meaning of FoxM1 interactions.

A total of 121 FoxM1 transcriptional targets were identified (Fig. 6), with 14 targets being reported to be experimental validated (*p*-value =2.33^−20^, Odds ratio = 75.95). Among the reported validated targets, 10 were reported to be involved in the curated FoxM1 transcriptional factor network according to the National Center of Bioinformatics Institute (NCBI) Biosystem Database (a source of all publication associated intersections in a specific biosystem). The other 4 targets were manually searched and identified in recent publications [7, 26, 35, 36]. Wonsey et al. used time-lapse microscopy to show that depletion of FoxM1 in basal cell carcinoma cells would generate cells that enter mitosis but were unable to complete cell division, resulting in either mitotic catastrophe or endoreduplication, confirming their hypothesis that FoxM1 might regulate genes essential for faithful chromosome segregation and mitosis, including NEK2, CENPA and KIF20A [35]. Lefebvre et al. showed experimentally that Aurora B Kinase (AURKA) functions by associating with the spindle poles to regulate entry into mitosis, centrosome maturation and spindle assembly as a FoxM1 target [7, 8].

Again, Wang et al. demonstrated that introducing FoxM1 siRNA resulted in reduction in both protein and mRNA level of AURKA and M-phase inducer phosphatase 1 (CDC25A), thus reinforcing the hypothesis that AURKA might be a direct transcriptional target of FoxM1 in breast cancer cell lines [36]. In another study, Martin et al. reported that Aurora A is tentatively associate with prognosis in ER+ tumors, which is different from the potential prognostic role of FoxM1 in ER- tumors [26]. Based on the inferred regulatory associations by GPU-ARACNE, it is feasible to speculate that the function of FoxM1 in ER+ might be compensated by the play-up of one of its direct targets, such as AURKA. To sum up, the FoxM1 regulon inferred by GPU-ARACNE was generally biologically validated according to the literature.

### Other GPU based implementations

There are at least three other implementations of gene regulatory network construction algorithms using GPU by Ramirez et al. [37], Borelli et al. [38], and Misra et al. [14], respectively. This signifies the trends and importance of parallelizing existing sequential algorithms for regulatory networks reconstruction. All of them were different from GPU-ARACNE in some aspects. Ramierz-Chavez et al. [37] inferred network using differential evolution algorithm, a genetic algorithm without using probability density function, achieving significantly time reduction. In Borelli et al. study [38], a feature selection procedure was used in the exhaustive search GPU paralleled algorithm, obtaining encouraging speedup. The most recent and so far the best performing parallel implementation was reported by Misra et al. [14]. In their work, they were able to estimate MI by fixed bandwidth, reporting that only hundreds of seconds were needed to compute pairwise MI values using Intel R Xeon Phi multiprocessor.

All those work contributed in different ways, varied greatly in terms of accuracy and computational complexity, making it difficult to directly compare their optimized implementations. Here, GPU-ARACNE is unique in that it highly optimizes adaptive partitioning in MI computation in CUDA framework. In our work, apMI estimator usually took less than a hundred seconds for one round of pairwise estimation. But, it should be noted that Misra et al. implemented on a different computing framework and on an old device. The upgraded GPU computing capacity aided the gain of speed in both GPU-ARACNE-V1 and GPU-ARACNE-V2. Besides, other large-scale technology such as MapReduce and Online database optimiztion can also be implemented to facilitate bioinformatics tasks [39, 40].

## Discussion

Here, we provide GPU-ARACNE, an accelerated parallel algorithm for reverse engineering of gene regulatory networks. GPU-ARACNE runs significantly faster than ARACNE and was comparably faster than the recently published multi-threaded JAVA version, ARACNE-AP. We provide two versions of GPU-ARACNE to ensure brand-agnostic compatibility with CUDA. GPU-ARACNE-V1 is applicable to any GPU with compute capability of 3.5 or higher. GPU-ARACNE-V2 is applicable to any GPU with compute capacity of lower than 3.5. The current Amazon Web Service provides readily usable platforms for those 2 versions, p2 instances for V1 and g2 instance V2.

The optimized GPU-based design of adaptive partitioning MI estimator provides valuable lessons that are applicable to other domains, such as higher order interactions or epigenome interactions. For example, besides transcriptional regulation, transcriptional modulation is also a very important biological process in cells. The interactions between transcriptional factor, transcriptional modulator, and gene target can be reconstructed based on mRNA expression using methods such as conditional mutual information (CMI [41]) and partial correlation. In the case of CMI estimation, one can extend the current splitting of 2D square into partitioning of 3D cube. Given the computational intensity of CMI estimation, GPU based re-design might be a very valuable attempt. Application of apMI to Chromatin binding factors and their targets would also bring merits to current field. For example, customizing apMI estimator to different data type, we might be able to largely improve the speed for algorithms [42].

By fully using GPU computation power (compute capability less than 3.5 or not), GPU-ARACNE was able to process a dataset up to 1024 samples without significantly degenerating the performance. In GPU-ARACNE implementations, SIMD instructions were used for parallel counting to optimize thread synchronization by reducing thread divergence. But still, computation will become very slow for datasets with sample size reaching hardware limit, possibly due to overuse of block-wise shared memory. Further explorations are needed to overcome this issue. For example, we can use mini-batch strategy, splitting original datasets into small ones to run, or splitting all regulators into sub-sets to run. Besides, using virtual warp in between GPU thread and block is also possible [43]. In this case, instead of using one thread for one data point, we can design a middle layer, so that one thread can process multiple data points, thus, the hardware limitation of thread number per block is going to be resolved.

Besides the hardware restriction, there is one caveat in applying GPU-ARACNE. If sample size is larger than the available blocks on GPU, GPU stream multiprocessors (SM) can only execute on a limited number of blocks according to GPU configuration, other blocks will be held for serialized executions. Thus, a careful calculation of GPU card theoretical upper bound is essential to minimize GPU sequential operation so that SM can execute a kernel in a greater parallel fashion. As we stored data for computing each edge in GPU block shared memory, the sample size is bounded by the stored data structure length (sample size - 3 in our case).

Also, a null model with closed-form solution is possible according to previous work [44] in which the author hypothesized connectivity matrix for inter dataset regulatory networks. Beyond current ARACNE framework, there are many other ways to achieve further parallelism, such as data level parallelism.

To explore further parallelism optimization possibilities on current GPU, we tried one thread per block so that SM can handle more blocks in computing each pairwise MI, hopefully increasing parallelism. However, it turned out slowing it down instead of accelerating up. This is possibly due to the fact that this re-design requires one thread to sequentially go through all samples before complete one apMI partition. Contrasting to the attempt, GPU-ARACNE wants each thread to be responsible only for one sample point, facilitating simultaneously counting for one apMI partition. It seems that we encountered a "two bottlenecks trade-off" situation in which either design is equivocally less ideal in some aspect. Practically, the sample size bottleneck seems to be less harmful for performance. First, it is promising that the block size limitation (threads limit in one block) might be mitigated with hardware improvement in the very near future. Also, in many cases GPU-ARACNE is used to construct regulatory networks using RNA-seq samples, for which sample size barely reaches the upper limit. Meanwhile, other optimization effort could help to improve GPU block usage, with more blocks taking advantage of fast memory access. Currently, each element in the node is 20 bytes in size, but by exploring the data we might possibly squeeze the informative bits of many numbers into one 64-bit number. Alternatively, instead of using one thread per point, one thread could potentially handle more points, and therefore increasing the throughput.

## Conclusions

ARACNE is one of most important algorithms for inference of regulatory networks from gene expression data, but it requires intensive computation. To achieve short execution time, researchers need to have access to expensive high-performance computing clusters, which are usually not readily available. GPU-ARACNE solved this problem by significantly reducing the computing time using GPU framework without losing the inference accuracy. Moreover, GPU-ARACNE exemplifies one of the first attempts to handle large-scale bioinformatics problems by parallel computing systems. It is characterized by exploiting different types of parallelism, including fine-grained and thread-level parallelism as well as data-level parallelism. The design and use of advanced parallel computing illustrated by GPU-ARACNE not only exhibit new possibilities to accelerate bioinformatics algorithms but also provide useful insights for importing other applications which might be beyond the scope of systems biology.

## Additional file

Additional file 1 Source code. The source code developed are available in the Bitbucket repository: https://github.com/califano-lab/GPU-ARACNE. (ZIP 25 kb)

## Abbreviations

apMI: Adaptive partitioning mutual information
ARACNE: Algorithm for the reconstruction of accurate cellular networks
ARACNE-AP: ARACNE using JAVA
AWS: Amazon Web Service
BRCA: Breast carcinoma
CMI: Conditional mutual information
CUDA: Compute unified device architecture
GPU: Graphics processing unit
GPU-ARACNE: GPU based ARACNE
JVM: JAVA virtual machine
MI: Mutual information
N_target_: Number of target genes
N_tf_: Number of transcription factors
N_sample_: Number of samples
PRAD: Prostate Adenocarcinoma
SIMD: Single instruction multiple data
SM: Stream Multiprocessor
